# Genomic Analysis of a Marine Bacterium: Bioinformatics for Comparison, Evaluation, and Interpretation of DNA Sequences

**DOI:** 10.1155/2016/7215379

**Published:** 2016-11-01

**Authors:** Bhagwan N. Rekadwad, Juan M. Gonzalez, Chandrahasya N. Khobragade

**Affiliations:** ^1^School of Life Sciences, Swami Ramanand Teerth Marathwada University, Nanded 431606, India; ^2^Institute of Natural Resources and Agrobiology, Spanish National Research Council, IRNAS-CSIC, Avda. Reina Mercedes 10, 41012 Sevilla, Spain

## Abstract

A total of five highly related strains of an unidentified marine bacterium were analyzed through their short genome sequences (AM260709–AM260713). Genome-to-Genome Distance (GGDC) showed high similarity to* Pseudoalteromonas haloplanktis* (X67024). The generated unique Quick Response (QR) codes indicated no identity to other microbial species or gene sequences. Chaos Game Representation (CGR) showed the number of bases concentrated in the area. Guanine residues were highest in number followed by cytosine. Frequency of Chaos Game Representation (FCGR) indicated that CC and GG blocks have higher frequency in the sequence from the evaluated marine bacterium strains. Maximum GC content for the marine bacterium strains ranged 53-54%. The use of QR codes, CGR, FCGR, and GC dataset helped in identifying and interpreting short genome sequences from specific isolates. A phylogenetic tree was constructed with the bootstrap test (1000 replicates) using MEGA6 software. Principal Component Analysis (PCA) was carried out using EMBL-EBI MUSCLE program. Thus, generated genomic data are of great assistance for hierarchical classification in Bacterial Systematics which combined with phenotypic features represents a basic procedure for a polyphasic approach on unambiguous bacterial isolate taxonomic classification.

## 1. Introduction

A wide range of microorganisms are isolated and identified using morphological, biochemical, and molecular features by numerous research groups worldwide. Bacterial differentiation and classification is a highly time-consuming procedure involving some ambiguous steps for the nonspecialist. Besides, most microorganisms on Earth are difficult or unable to be cultured and barely 1% of all the expected different microorganisms on our planet have been properly described [1, 2]. Recent views of the microbial community structures in nature and artificial complex environments highlight the huge diversity of microorganisms forming these communities. In fact, microbial diversity is, at present, hard to determine and numerous efforts are carried out on this hot topic [3, 4]. The only methodological strategy to face this enormous microbial diversity is the use of DNA sequencing procedures and the consequent bioinformatic analyses.

In the proposed research paper an attempt has been made to digitize the marine bacterium data for the first time using short DNA sequences (16S rDNA sequences). Obtained DNA sequences are used to confirm identity of bacterial species amongst the known sequences available in databases, such as NCBI-BLAST and Ribosomal database. While identifying microorganisms using the conserved DNA sequence, mistakes frequently occur due to large number of hits with minute differences. Additionally, at present, the use of sequencing technology often lacks tools for visual interpretation and comparison of DNA sequences with other cells from the environment. To overcome this problem DNA digitalization including QR code, CGR, FCGR, GC content, and PCA are proposed as potential satisfying tools for the study of genes/DNA sequences. The proposed pipeline represents a standardizable, fast, and reliable tool for identification of microorganisms up to the species level using DNA sequences.

## 2. Materials and Methods

The FASTA format sequences of identified bacteria (AM260709–AM260713) were retrieved from NCBI repository. These correspond to five highly similar marine bacterium isolates which we will attempt to unambiguously differentiate. Genome-to-Genome Distance Calculator tool, DSMZ (http://ggdc.dsmz.de/distcalc2.php), was used for calculation of DNA to DNA difference [5]. The DNA QR codes of identified bacterial species were generated using DNA BarID tool, http://www.neeri.res.in/DNA_BarID/DNA_BarID.html. The generated QR codes for the species do not resemble any other species or strains in any database. Any user can scan these QR codes and read more information on bacterial species. This information is useful to identify and compare the QR-coded isolates or sequences. The generated data were compared with other visual techniques such as CGR and FCGR. GC contents of the marine bacteria were determined using ENDMEMO GC calculating and GC plotting tool [6, 7]. The phylogenetic tree was constructed using MEGA6. The evolutionary history was inferred using the Neighbor-Joining method. The optimal tree with the sum of branch length equaling 2.34244687 is shown. The percentage of replicate trees in which the associated taxa clustered together in the bootstrap test (1000 replicates) is shown next to the branches. The tree is drawn to scale, with branch lengths in the same units as those of the evolutionary distances used to infer the phylogenetic tree. The evolutionary distances were computed using the Maximum Composite Likelihood method and are in the units of number of base substitutions per site. The analysis involved 5 nucleotide sequences. All positions containing gaps and missing data were eliminated. There were a total of 1480 positions in the final dataset. Evolutionary analyses were conducted in MEGA6 [8–12]. Principal Component Analysis (PCA) was carried out using multiple alignment program EMBIL-EBI MUSCLE (http://www.ebi.ac.uk/Tools/msa/muscle/) for comparative analysis [13–15].

### 2.1. Data Summary

DNA sequence data for marine bacterium (accession nos.: AM260709–AM260713) are available via the NCBI repository (http://www.ncbi.nlm.nih.gov/nuccore).

## 3. Results

**Figure 1:**
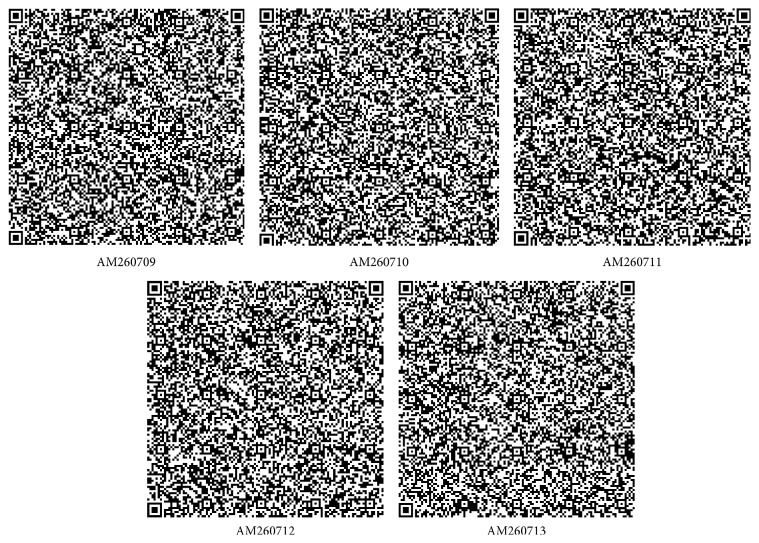
QR codes of marine bacterial 16S rRNA gene sequences: AM260709–AM260713.

**Figure 2:**
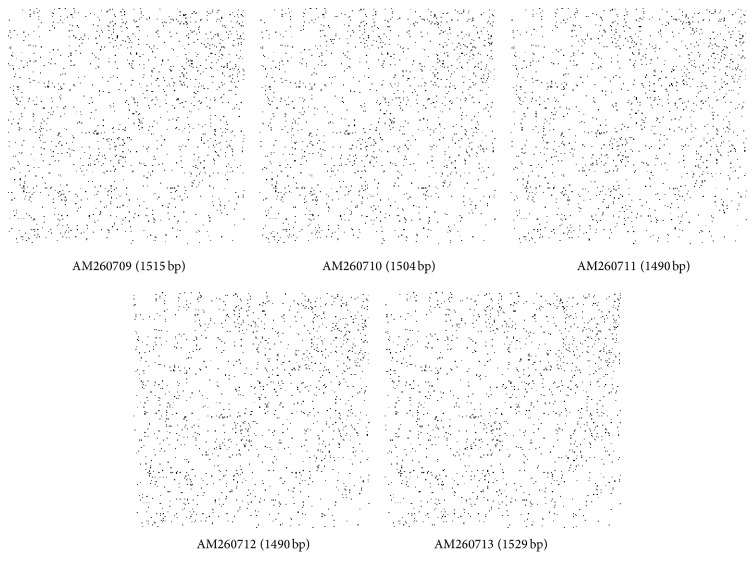
CGR of marine bacterial sequences: AM260709–AM260713.

**Figure 3:**
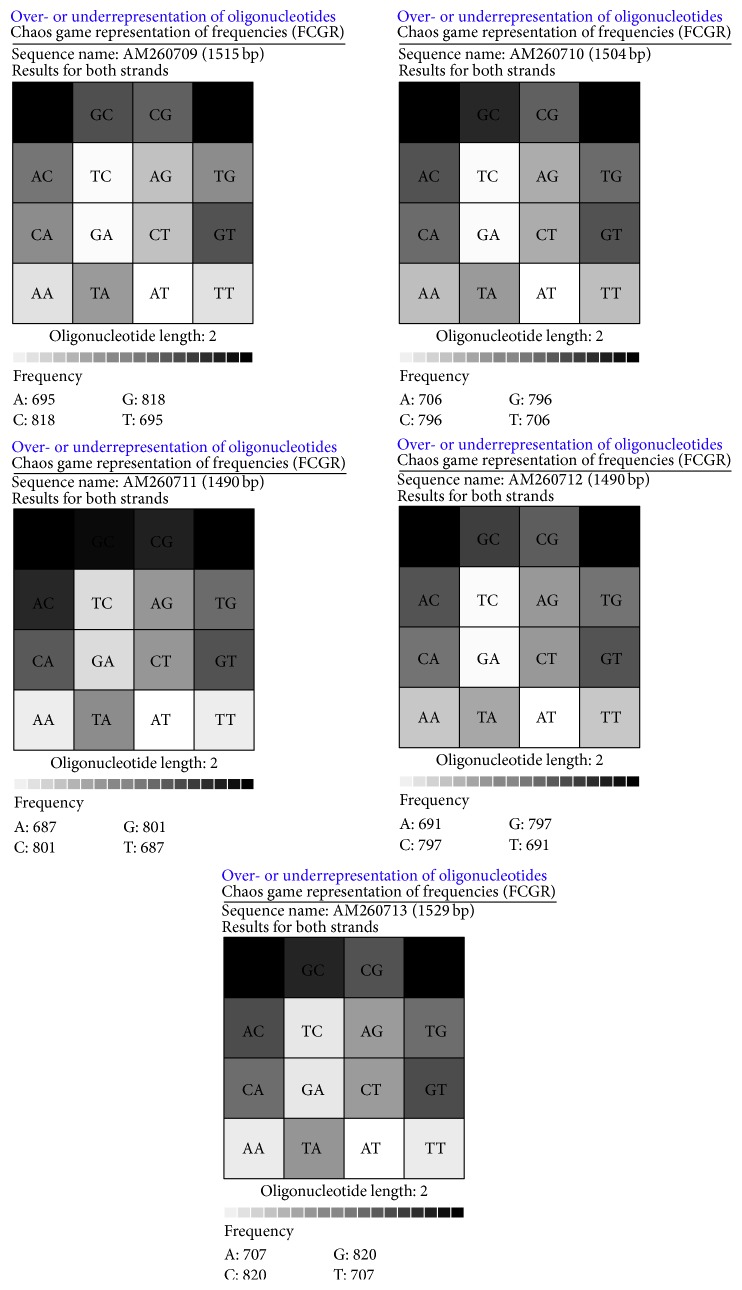
FCGR of marine bacteria for sequences: AM260709–AM260713.

**Figure 4:**
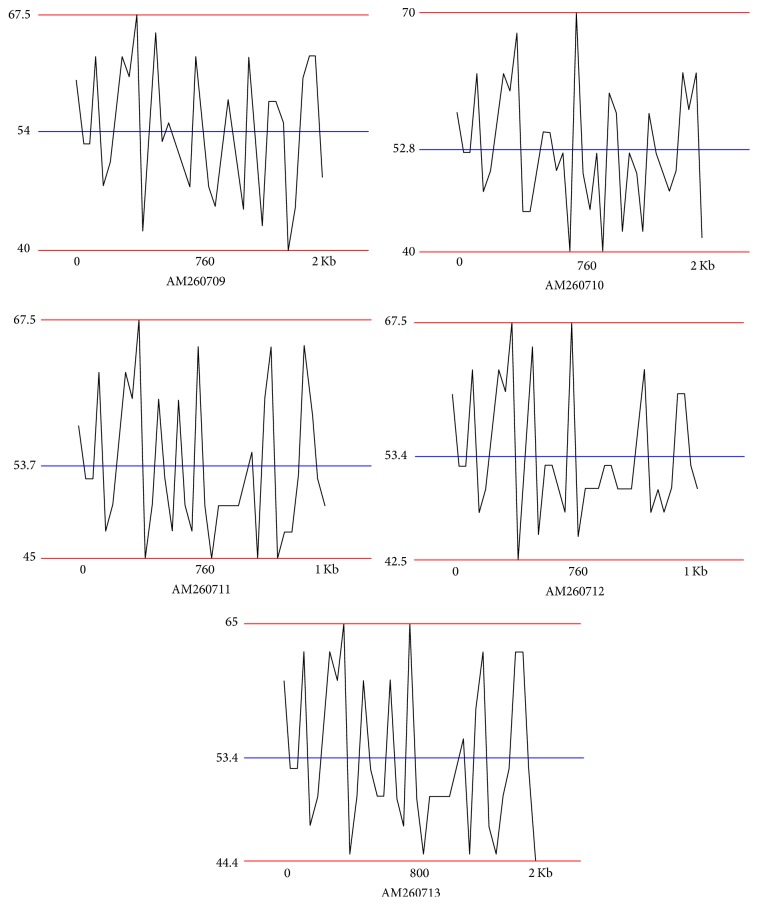
GC plots of marine bacterial sequences: AM260709–AM260713.

**Figure 5:**
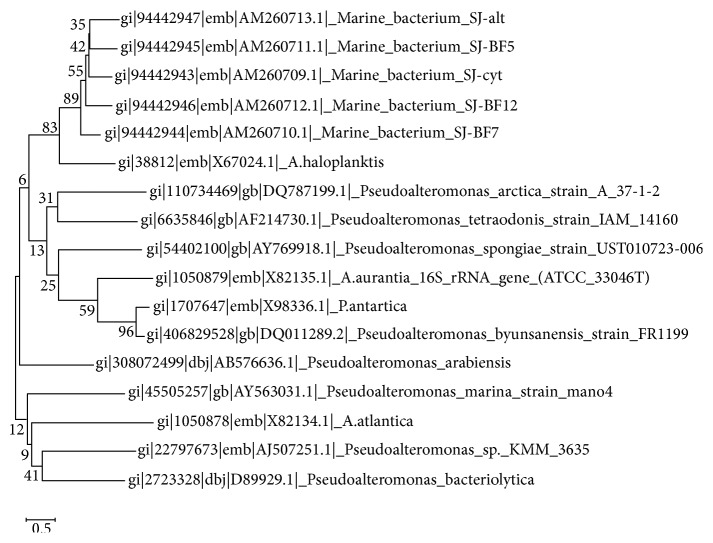
Evolutionary relationships amongst the evaluated marine bacteria (sequences AM260709–AM260713) showing three lineages and the differentiation of the distinct strains.

**Figure 6:**
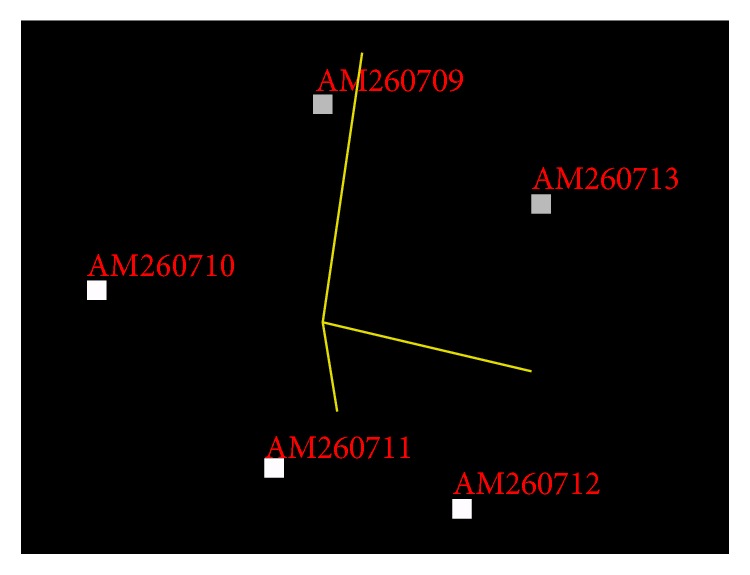
Principal Component Analysis of marine bacterial strains (AM260709–AM260713) showing clear differentiation of the studied marine bacterium strains forming a phylogenetically close group of bacteria.

A total of five strains of marine bacterium short genome sequences (AM260709–AM260713) were retrieved from NCBI BioSample repository. Genomic BLAST revealed that all marine bacterium species showed 99% similarity with each other. The percentage of similarity of marine bacterium (AM260709–AM260711 and AM260713) with* Pseudoalteromonas haloplanktis* (X67024) is 97%. AM260712 showed 98% similarity with* P. haloplanktis* in NCBI taxonomic database. The Genome-to-Genome Distance (GGDC) results clearly indicated that all marine bacterium showed 70% DDH similarity with* P. haloplanktis*. The differences between DDH values between marine bacterium and* P. haloplanktis* were ranged from 0.06% to 1.01% (Table 1). The unique quick response (QR) codes for each strain generated did not show identity with any other species or gene sequences in any database (Figure 1). Each QR code occupies 2 kb to 65 kb in the computer memory space which is similar to the space occupied by DNA sequences. Chaos Game Representation (CGR) drawn and appearance of bases in the graphical representation were studied. It was visually observed that a higher number of bases concentrated on the G corner of the CGR image (Figure 2). It was observed that guanine residues were higher in number followed by cytosine. The appearance of Frequency of Chaos Game Representation (FCGR) was recorded. FCGR image has a unique pattern of distribution of nucleotides in the blocks. Increase in dark color in the block is directly proportional to the number of nucleotides. It was represented that CC and GG blocks in FCGR were darker and have higher frequency of repetition in the area in all tested marine bacterium strains (Figure 3). GC contents of marine bacterium strains were determined using ENDMEMO GC calculating and GC plotting tool using short DNA sequences. Upper and lower red lines indicate maximum and minimum GC content in the given plot. Similarly middle blue line indicates the average GC percentage in the given short DNA sequence. Maximum GC content was observed in marine bacterium species average 53-54% and ranged 40–70% (Figure 4). The use of QR codes, CGR, FCGR, and GC dataset helps to identify and interpret short genome sequence of isolates. The QR codes were generated using sequences. The darkness of QR codes and appearance of mosaic spot reflect the differences amongst them. The visual data of QR codes in the form of images can be scanned using any smartphone containing QRStuff tool (http://www.qrstuff.com/qr_phone_software.html) and compared for presence of different nucleotides. Both CGR and FCGR can be interpreted and compared visually. Each CGR image has four corners (C, G, A, and T) forming four equal squares in each image. The number of dots appearing in each subsquare is directly proportional to the number of nucleotides appearing in it. The number of dots is equal to the number of nucleotides. In FCGR image, a color tape is given. The color tape has lighter color indicator changing to darker and darkest color. The 12 subsquares in each FCGR were not the same. The color of each square is directly proportional to the number of base pairs/nucleotides. Lighter color indicates few nucleotides while darkest color indicates more numbers of nucleotides. It can be compared with color tape provided with each FCGR image. The phylogenetic tree was constructed amongst the marine bacterium strains under study using MEGA6 software. The bootstrap test was carried out using 1000 replicates (Figure 5). It is observed that marine bacterium sequences showed more similarity about more than 97% with* Pseudoalteromonas haloplanktis* (*A. haloplanktis*).* Pseudoalteromonas* species in each clad showed identity with marine bacterium. This reveals and confirms the similarity between marine bacterium and typical* Pseudoalteromonas* species. Principal Component Analysis (PCA) was carried out using EMBL-EBI MUSCLE program. It was observed that all marine bacterium strains except marine bacterium strain SJ-alt (AM260713) fall in another plane and show slight differentiating sequence compared to the rest of the strains with sequences AM260709–AM260712 (Figure 6, Table 2). Hence, the present digital data probe to be highly useful to investigate differences amongst very similar strains. Thus, generated genomic digital data help for higher level hierarchical classification in Bacterial Systematics along with unambiguous differentiation of closely related isolates.

### 3.1. Impact Statement

Digital data (i.e., digitalization of data obtained from DNA sequencing) act as limelight for identification, exactness, and comparison of new isolated marine bacterium species. At present, the differentiation of closely related bacteria and the quick bacterial identification are present major gaps and represent areas of high demand of novel initiatives for simplified and quick procedures. Digital data would be valuable for quantitative and qualitative analyses of newly isolated microorganisms, for example, the case of marine bacterium strains. At present, these generated data represent a baseline to any researcher by economics, rapidity, and ease of obtaining. Herein, we propose an original analysis pipeline which includes a novel bioinformatics approach generating digital data from DNA sequencing information. This is applied to the case of marine bacterium strain differentiation and classification.

## 4. Discussion

This study provides a digital dataset for the identification, comparison, evaluation, and interpretation of newly isolated strains from the environmental samples. Differentiation of bacteria obtained from an environment results in a relatively complicated task when those bacteria are phylogenetically closely related and is even more problematic when one is dealing with so far unclassified bacteria (which are abundant in noncurated public repositories). An easy differentiating pipeline would be greatly useful for a large number of applications including natural environments and man-generated habitats such as the clinical or industrial scenarios. The type of data generated in this study can be produced for any prokaryotic species using the protocol herein described. As well, similar types of data and analyses may be performed on eukaryote sequence data. Overall, the enlisted data and protocol will be useful to research and industry in a very broad range of disciplines. The proposed pipeline for the digital short sequence data comparisons solves the problem of identification and/or differentiation of newly detected microorganisms, above all, those closely related amongst them, either previously described or unclassified microorganisms. The method can be directly applied to be used with any gene, set of genes, or DNA fragments of variable nature and length. Thus, this novel approach can increase its specificity and applicability as needed. This will be a function of the DNA sequences introduced in the pipeline for analysis.

## Supplementary Material

The supplementary file contains GGDC results are supplied with this paper. It shows DNA-DNA Hybridisation (DDH) data.

## Figures and Tables

**Table 1:** Genome-to-Genome Distance calculation: marine bacterium versus *Pseudoalteromonas haloplanktis* (X67024).

Accession number	DNA-DNA hybridization (DDH)	GC content difference in DDH (marine bacterium versus *P. haloplanktis*)	% Similarity in genomic BLAST with *P. haloplanktis*
AM260709	70%	1.01	97%
AM260710	70%	0.06	97%
AM260711	70%	0.78	97%
AM260712	70%	0.51	98%
AM260713	70%	0.65	97%

**Table 2:** Molecular characteristics of marine bacterium short DNA sequences.

Accession number	Strain	Sequence length (bp)	GC (%)	Molecular weight (MW) in Dalton
AM260709	SJ-cyt	1515	54	496133.28
AM260710	SJ-BF7	1504	53	492275.04
AM260711	SJ-BF5	1490	54	487791.15
AM260712	SJ-BF12	1490	53	487905.29
AM260713	SJ-alt	1529	54	500706.13
